# New Formulations Loading Caspofungin for Topical Therapy of Vulvovaginal Candidiasis

**DOI:** 10.3390/gels7040259

**Published:** 2021-12-12

**Authors:** Noelia Pérez-González, Nuria Bozal-de Febrer, Ana C. Calpena-Campmany, Anna Nardi-Ricart, María J. Rodríguez-Lagunas, José A. Morales-Molina, José L. Soriano-Ruiz, Francisco Fernández-Campos, Beatriz Clares-Naveros

**Affiliations:** 1 Department of Pharmacy and Pharmaceutical Technology, Faculty of Pharmacy, Campus of Cartuja, University of Granada, 18071 Granada, Spain; noeliaperez93@correo.ugr.es (N.P.-G.); annanardi@ub.edu (A.N.-R.); jlsoriano@correo.ugr.es (J.L.S.-R.); beatrizclares@ugr.es (B.C.-N.); 2 Department of Biology, Healthcare and the Environment, Faculty of Pharmacy and Food Sciences, University of Barcelona, 27-31 Joan XXIII Ave., 08028 Barcelona, Spain; nuriabozaldefebrer@ub.edu; 3 Department of Pharmacy and Pharmaceutical Technology and Physical Chemistry, Faculty of Pharmacy and Food Sciences, University of Barcelona, 27-31 Joan XXIII Ave., 08028 Barcelona, Spain; anacalpena@ub.edu; 4 Institute of Nanoscience and Nanotechnology (IN2UB), University of Barcelona, 08028 Barcelona, Spain; 5 Department of Biochemistry and Physiology, Faculty of Pharmacy and Food Sciences, University of Barcelona, 08028 Barcelona, Spain; mjrodriguez@ub.edu; 6 Institute of Biomedicine, University of Barcelona, 08028 Barcelona, Spain; 7 Department of Pharmacy, Torrecárdenas University Hospital, s/n Hermandad de Donantes de Sangre St., 04009 Almeria, Spain; joseantonio.morales@ephpo.es; 8 Reig-Jofre Laboratories, Av. de les Flors s/n, 08970 Sant Joan Despi, Spain; 9 Biosanitary Institute of Granada (ibs.GRANADA), 18012 Granada, Spain

**Keywords:** vulvovaginal candidiasis, antifungal therapy, caspofungin, vaginal mucosal delivery, mucoadhesive hydrogel, thermoreversible hydrogel

## Abstract

Vulvovaginal candidiasis (VVC) poses a significant problem worldwide affecting women from all strata of society. It is manifested as changes in vaginal discharge, irritation, itching and stinging sensation. Although most patients respond to topical treatment, there is still a need for increase the therapeutic arsenal due to resistances to anti-infective agents. The present study was designed to develop and characterize three hydrogels of chitosan (CTS), Poloxamer 407 (P407) and a combination of both containing 2% caspofungin (CSP) for the vaginal treatment of VVC. CTS was used by its mucoadhesive properties and P407 was used to exploit potential advantages related to increasing drug concentration in order to provide a local effect. The formulations were physically, mechanically and morphologically characterized. Drug release profile and ex vivo vaginal permeation studies were performed. Antifungal efficacy against different strains of *Candida* spp. was also evaluated. In addition, tolerance of formulations was studied by histological analysis. Results confirmed that CSP hydrogels could be proposed as promising candidates for the treatment of VVC.

## 1. Introduction

Vulvovaginal candidiasis (VVC) is a health problem that affects approximately 70–75% of women worldwide at least once during their lifetime [1,2]. It has been estimated that about 50% of women will suffer VVC again and 8% will develop chronic VVC [3]. The symptoms include changes in vaginal discharge, irritation and itching sensation, stinging, burning sensation inside and/or outside the vagina, pain during urination and discomfort during sexual intercourse. Other symptoms include scratching lesions, swelling and vulvar redness [4]. Candidiasis is commonly caused by *Candida* spp. The predominant species are *C. albicans*, followed by *C. glabrata*, *C. tropicalis*, *C. krusei* and *C. parapsilosis* [5]. VVC is estimated to be the second most common cause of vaginitis after bacterial vaginosis. *C. albicans* accounts for 85–90% of cases [6].

On the other hand, infections of the genitourinary system are the second reason for consultation during and after the severe acute respiratory syndrome coronavirus 2 (SARS-CoV-2) pandemic. Without epidemiological studies to confirm this, a considerable portion of health care has been motivated by vulvovaginal symptoms compatible with vulvovaginitis due to *Candida* spp. Changes in habits, stress, the use of broad-spectrum antibiotics or dexamethasone [7], prolonged stays in the intensive care units, or catheters may be important factors that cause patients with COVID-19 to develop these vaginal infections through the alteration of the microbiological balance of the area [8]. In addition, COVID-19 disease, caused by SARS-CoV-2, may increase systemic fungal co-infections associated with COVID-19. Patients with COVID-19, especially those who are affected severely or immunocompromised are more susceptible to develop invasive mycoses [9].

Although most patients with VVC respond to topical treatment, mortality rates associated with invasive fungal infections are really high, and there is a growing concern about resistance to anti-infective agents [10,11]. Fortunately, the arsenal of antifungal agents is continuously improved and a new antifungal drug family is being developed: echinocandins [12,13,14]. Among this family caspofungin (CSP) is a semi-synthetic water soluble lipopeptide obtained from *Glarea lozoyensis* as a fermentation product. The mechanism of action consists of blocking the synthesis of an essential component of the cell wall of numerous fungal species, β (1,3)-D-glucan. CSP is presented as a lyophilized powder in its acetate form. This drug is only available for hospital use and it is administered intravenously [15,16,17,18].

It is of outmost importance for drug administration through vaginal area to take into account the anatomy of this especial cavity as well as its secretions, as the resulting fluids can alter both the retention of the formulation and the release of the drug [19]. To overcome these problems, formulations aimed to provide spatial and temporal control of drug delivery have been designed by providing coverage of the affected areas without elimination, as well as being compatible with aqueous environments. These formulations are hydrogels [20]. Different hydrogels as drug delivery system against VVC have been used focusing on the effective therapeutic response at the vaginal mucosa. A review of the literature shows that most of these formulations are hydrogels loaded with classical antifungal drugs such as amphotericin B, miconazole or clotrimazole [21,22,23,24,25]. However, other authors have opted to incorporate into their formulations alkannin, diphenyl diselenide, anise or terbinafine, among others [26,27,28,29]. There are not previous studies reporting the delivery of CSP because is the first time it has been applied topically in vaginal mucosa. Only a topical study of eye drops has been found in the literature [30].

It is well-known that hydrogels are three-dimensional polymeric networks containing large amounts of water in their structure [31]. Water-soluble polymers have the ability to adhere to mucosal tissues. These mucoadhesive properties are related to those charged polymers with a higher molecular weight, which possess greater ability to adhere to tissues [32]. Chitosan (CTS) is a cationic polysaccharide that exhibits antimicrobial activity. It is also characterized by mucoadhesive properties [33,34,35]. CTS has been chosen in this work because of its biocompatibility, biodegradability, ability to form gels in situ with negatively charged macromolecules and for its mucoadhesive, antimicrobial and antifungal properties. The cationic nature of CTS confers a sol-gel transition at pH 6.5, when the medium changes from acidic to neutral [36,37]. In its context, poloxamer 407 (P407) is a hydrophilic non-ionic surfactant belonging to the poloxamers copolymers family, which are known for being thermoreversible [38]. Both CTS and P407 have been widely used as delivery systems for the release of different drugs for vaginal administration [39,40,41,42,43].

In recent years, polymer combination designs have been developed to give them multifunctionality. Natural polymers, such as CTS, ensure minimally invasive gels in the human body, while synthetic polymers, such as P407, produce gels with high mechanical resistance. The advantage of joining both polymers would let us to obtain a hydrogel with optimal biocompatible and biodegradable nature, high-water content, high responsiveness and permeability, and then obtain an improved therapeutic effect [44]. Both polymers are linked by non-covalent interactions between the amino groups of CTS and the ethylene oxide units of P407 to form a high-strength physical [45]. The combination of these polymers has also been used for drug delivery, including vaginal administration [46,47,48,49].

Thus, the main objective of this study is the development of a topical delivery system for the vehiculization of CSP. The vaginal topical use of CSP has never been reported. For this general purpose, three formulations in form of hydrogels of CTS, P407 and the combination of both have been investigated for the treatment of VVC. The specific goals of this research are: (i) the detailed physicochemical characterization of CSP hydrogels for topical application, (ii) the in vitro release and ex vivo permeation studies through vaginal tissues, (iii) the in vitro studies of antifungal activity and, (iv) the histological evaluation of the effect of these formulations.

## 2. Results and Discussion

### 2.1. Physical Characterization of Caspofungin Hydrogels

#### 2.1.1. pH Evaluation

The pH values for CSP-CTS, CSP-P407 and CSP-CTS/P407 hydrogels were 4.58 ± 0.05, 6.27 ± 0.03 and 4.24 ± 0.05, respectively. None of these values changed after three month-storage period at 4 ± 1.00 °C. When the formulations were mixed with simulated vaginal fluid (SVF), the pH of CSP-CTS and CSP-CTS/P407 remained unaltered, while the pH of CSP-P407 decreased to 4.58 ± 0.10.

Although the pH of the vagina is around 4.5, it can be affected when an infection occurs, then it could reach up to 7 [10,50]. In the present study, the pH of the formulations was found to be within the vaginal pH conditions.

#### 2.1.2. Morphological Studies

**Figure 1:**
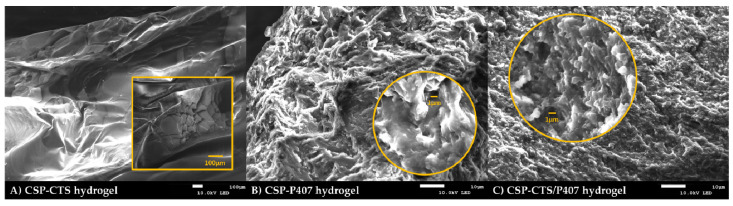
Scanning Electron Microscope micrographs images obtained for: (**A**) CSP-CTS hydrogel at 50× (magnification 850×); (**B**) CSP-P407 hydrogel at 1200× (magnification 4300×); and (**C**) CSP-CTS/P407 hydrogel at 1200× (magnification 4500×).

In order to characterize the microstructural architecture of samples a Scanning Electron Microscopy (SEM) analysis was performed. Figure 1A shows a uniform network sheet of CSP-CTS hydrogel without pores or cracks in its fibrous structure. As a result, a homogeneous and well-controlled final morphology has been obtained. The figure corresponding with the sample CSP-P407 hydrogel (Figure 1B) illustrates a highly porous structure typical of P407. Figure 1C shows a highly porous network with more distinct pores than CSP-P407 network, resulting in better interconnection between CTS and P407 [51]. Probably, for CSP-P407 hydrogel micelles contacted mutually similarly to the increase in viscosity. Instead for the CSP-CTS/P407 hydrogel chitosan is sandwiched between micelles [52].

Finally, the SEM image confirms that the drug has been correctly incorporated into the hydrogels because no drug crystals were observed adhering to the surface.

#### 2.1.3. Surface Electrical Properties

The surface charge of the formulation is a parameter that informs about its stability, clearance, as well as its solubility and drug distribution [53]. As shown in Table 1, the zeta potential (ZP) values were 80.36 ± 4.72, 11.14 ± 1.62 and 49.76 ± 3.22 mV for CTS, P407 and CTS/P407 hydrogels, respectively. Values for CSP loaded hydrogels were 74.00 ± 4.19, 9.94 ± 0.74 and 40.51 ± 3.87 mV for CSP-CTS, CSP-P407 and CSP-CTS/P407 hydrogels, respectively. No significant changes (*p* < 0.05) in ZP values were observed when CSP was incorporated into the formulations, indicating that it was uniformly distributed in the system without precipitation. 

#### 2.1.4. Rheological Behavior

The rheological characteristics of formulations play an important role in physical stability, sensorial characteristics, spreadness and dosing behavior. All these properties are related with the application characteristics and are crucial attributes for the development of topical drug products [54,55,56]. Sample readings were taken at 4, 25 and 37 °C in order to reproduce the behavior after storage in the refrigerator or at room temperature, and after application in the vaginal cavity.

Under rotational testing, CSP-CTS and CTS/P407 hydrogels presented a non-newtonian behavior (Table 2), adjusting to a shear thinning (pseudoplastic) profile, being the Cross equation the model that best fitted experimental data. Moreover, the flow curves showed an apparent thixotropic behavior in both systems as the rheograms displayed a hysteresis loop. These rheological characteristics are desirable for topical application, allowing the formation of a stable and consistent film covering the application area that allows the diffusion of the drug through the matrix [54,57,58]. This can also be an interesting feature because it improves syringeability properties and thus facilitate vaginal administration with the cannula.

The results of Table 2 also reveal that CSP-CTS hydrogel presented at 50 s^−1^ higher viscosity values than CSP-CTS/P407 hydrogel, 2237 ± 13.40 mPa·s and 1868 ± 14.11 mPa·s at 4 °C, respectively. For both formulations, the increase of temperature involved similar decrease in the viscosity under rotational testing conditions. However, the CSP-P407 hydrogel corresponds to a typical poloxamer-based thermoreversible gel which has the characteristic of being liquid and syringeable at low temperatures and gel at the body temperature [59]. Therefore, the CSP-P407 hydrogel viscosity increased from 16.45 ± 0.60 mPa·s (SOL structure at 4 °C) to 70.73 ± 2.02 mPa·s (GEL structure at 37 °C). These viscosity values were much lower than those obtained for gels CSP-CTS hydrogel and CSP-CTS/P407 hydrogel, both with the same quantity of CTS. In parallel, the CSP-P407 hydrogel flow curve presented a nearly newtonian behavior at 4 °C and a shear thinning character at 37 °C, according to the change of the structure of the system. 

**Figure 2:**
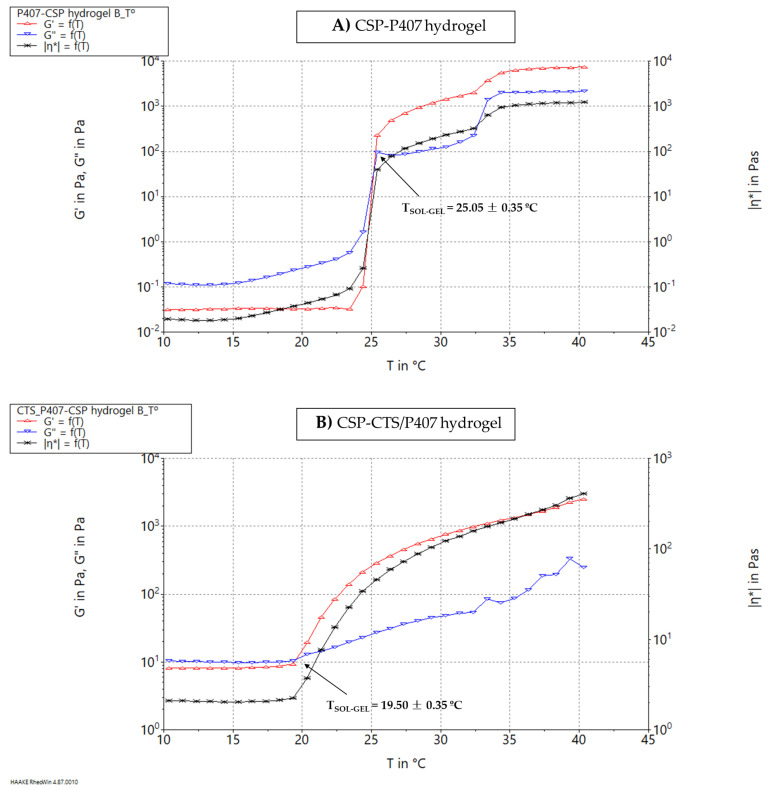
SOL-GEL Temperature Transition: (**A**) CSP-P407 hydrogel; and (**B**) CSP-CTS/P407 hydrogel.

Although the rotational testing did not detect the thermogelling phenomenon of the poloxamer included in the combination of CSP-CTS/P407 system, the temperature sweep under oscillatory testing did give evidence of this process. The results of the temperature sweep test performed to investigate the in-situ poloxamer gelling in the case of CSP-P407 hydrogel and CSP-CTS/P407 hydrogel are depicted in Figure 2. According to the CSP-P407 hydrogel graph (Figure 2A), the T_SOL-GEL_ (where G′ = G″) was about 25 °C. This is the temperature at which the sample exhibits a switch from a prevalently viscous behavior (G″ > G′) to a prevalently elastic one (G′ > G″). This interesting gelation process results in an increase in the residence time avoiding the rapid gel elimination [59]. 

Regarding the CSP-CTS/P407 hydrogel graph (Figure 2B), the crossover corresponding to the T_SOL-GEL_ took place earlier, at 19.5 °C and compared to the CSP-P407 hydrogel graph, at low temperatures the values of both G’ and G’’ moduli were much higher. It has been indicated that using a 20% (*w*/*v*) solution of poloxamer, with the addition of chitosan (1.5% *w*/*v*) a syringeable, thermosensitive and mucoadhesive gel could be obtained [60]. However, in the case of CSP-CTS/P407 hydrogel, the addition of CTS modifies the properties of the system. CTS interacts with P407 through its polyethylene oxide chains, increasing the number of hydrophilic chains and therefore the thickness of the micellar crown. Due to this interaction, the apparent viscosity could increase and the temperature T_SOL-GEL_ could decrease [52,61]. 

#### 2.1.5. Spreadability Test

**Figure 3:**
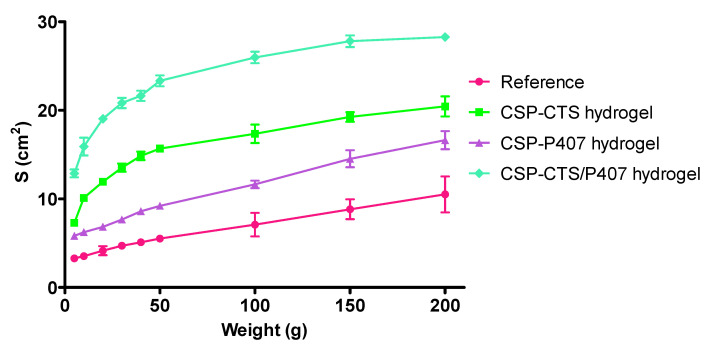
Spreading area (S, cm^2^) as a function of the applied mass (g) at 37 °C of reference and CSP-CTS, CSP-P407 and CSP-CTS/P407 hydrogels. Each value represents the mean ± SD (*n* = 3).

Spreadability properties of samples are important parameters to evaluate in topical forms because can influence both the packaging process and the uniformity of dose and application, and therefore the therapeutic efficacy [57,62]. The data obtained in the test are presented in Figure 3. It can be easily observed that the developed hydrogels exhibit higher extensibility values than the reference sample. CSP-CTS/P407 hydrogel possess the highest area, 28.27 cm^2^, followed by CSP-CTS and P407-CPS hydrogels, with areas of 20.44 and 16.63 cm^2^, respectively. The reference sample showed an area of 10.51 cm^2^.

In terms of application, CSP-CTS/P407 hydrogel facilitates application on the vaginal mucosal, an anatomical area where application is especially difficult.

**Figure 4:**
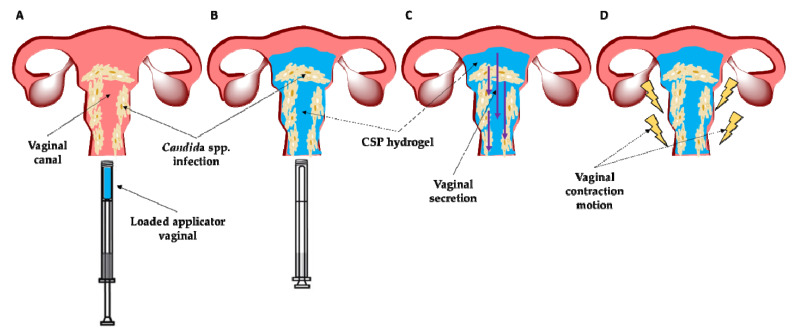
Schematic illustration of the mechanism of hydrogel application: (**A**) Administration into the vaginal cavity of the formulation through an applicator; (**B**) Expansion of the hydrogel through the vaginal cavity covering the infection focus; (**C**) Resistance of the hydrogel to vaginal secretion; and (**D**) Resistance of the hydrogel to vaginal contraction movement.

Promisingly, CSP-CTS/P407 hydrogel can be a potential formulation for the treatment of vaginal infection. Due to its thermo-sensitive properties, the hydrogel in the SOL state penetrate to the infection sites in deep vaginal rugae, in contrast in GEL state will provide improved mucosal adhesion and prolonged drug retention (Figure 4). 

#### 2.1.6. Extrudability Study

The results of extrudability can be observed in Table 3. It should be taken into consideration that vaginal applicators are hollow tubes whose lumen is much bigger than that of a syringe. Therefore, it would be convenient for the formulation to be expelled by exerting the minimal force as possible [57]. 

As might be expected, CSP-P407 hydrogel is the sample that extrudes most easily, but this could cause administration problems due to spillage. The reference sample has the highest extrusion value, indicating that it requires the maximum force to be extruded from the applicator. In contrast, CSP-CTS/P407 hydrogel shows better extrudability than CSP-CTS hydrogel. Based on these results, the hydrogel formed by the combination of both polymers shows the best extrudability properties.

#### 2.1.7. Evaluation of Mucoadhesion Properties

**Figure 5:**
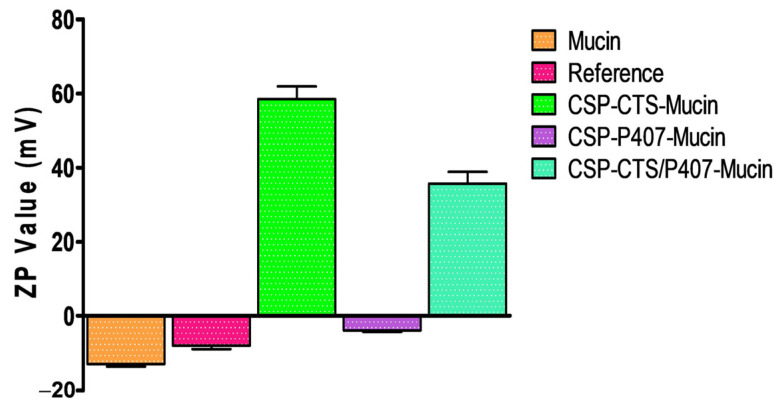
Zeta potential of mucin solution, CSP-CTS-Mucin solution, CSP-P407-Mucin solution and CSP-CTS/P407-Mucin solution Results are expressed as mean values ± SD (*n* = 9).

The mucoadhesive properties of vaginal formulations are essential due to the self-cleaning of the vagina. When compared the ZP data of the mucin solution with the ZP data obtained for the hydrogel-mucin mixtures, it was observed that the ZP value of the mucin dispersion, −12.86 ± 0.70 mV, changed when mixed it with the samples as shown in Figure 5. This change in value would be due to the interactions of the negative groups, such as carboxylate (COO^−^) and sulfonate (SO_3_^−^) of mucin with the positive groups of the polymers [63]. A polymer with mucoadhesive properties is able to neutralize the mucin charge [64]. The hydrogel that showed the maximum displacement was CSP-CTS, 58.47 ± 3.46 mV, confirming the mucoadhesive properties attributed to CTS due to its cationic nature [65], followed by CSP-CTS/P407, the reference and CSP-P407 with ZP values of 35.59± 3.15, −7.92 ± 0.98 and −3.89 ± 0.36 mV, respectively.

### 2.2. In Vitro Release and Kinetic Evaluation

**Figure 6:**
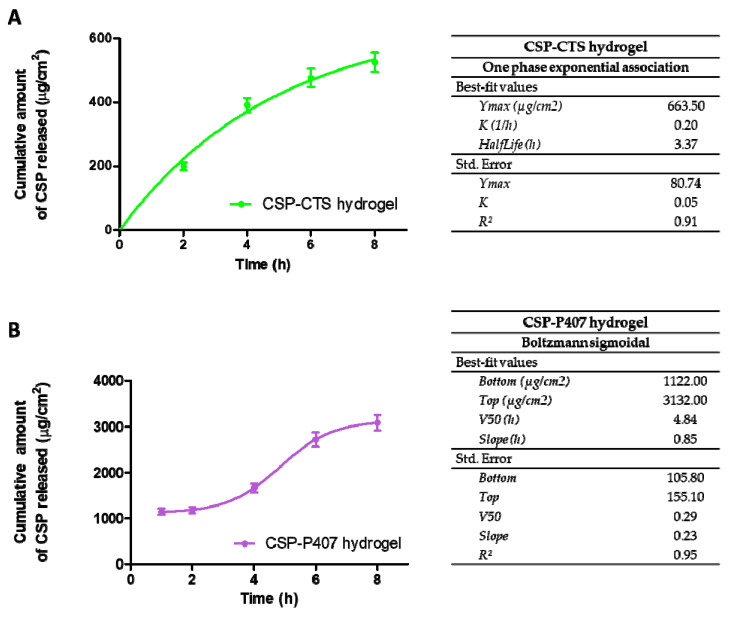

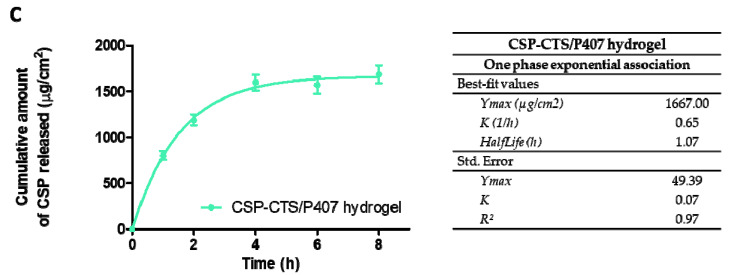
Cumulative amounts of CSP released from: (**A**) CSP-CTS hydrogel at 37 °C; (**B**) CSP-P407 hydrogel at 37 °C; and (**C**) CSP-CTS/P407 hydrogel at 37 °C. Data represent mean ± SD (*n* = 3).

The release profiles of the CSP hydrogels can be observed in Figure 6. After 8 h, the highest cumulative drug release was registered for CSP-P407 hydrogel. Its micellar properties provide a system with excellent solubility [66]; followed by CSP-CTS/P407 and CSP-CTS. 

The results of model fitting showed different release profiles for samples. CSP-CTS and CSP-CTS/P407 were adjusted to a Fickian kinetic model while CSP-P407 was adjusted to a sigmoidal model. Based on each equation, the release rates for CSP-CTS, CSP-P407, and CSP-CTS/P407 were 0.20 h^−1^, 0.85 h and 0.65 h^−1^, respectively.

**Figure 7:**
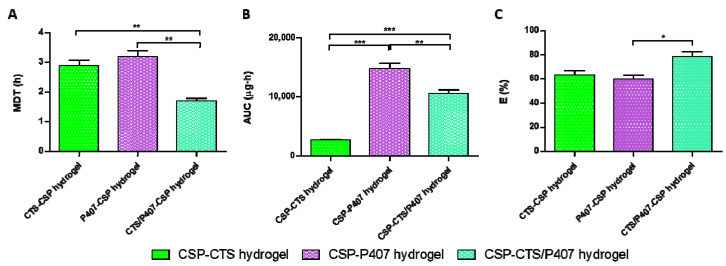
Non-modelistic parameters of: (**A**) mean dissolution time, MDT; (**B**) area under the curve, AUC; and (**C**) efficiency, E (* *p* < 0.05, ** *p* < 0.01, *** *p* < 0.001).

In order to compare the biopharmaceutical profile of all formulations, some amodelistic parameters (Figure 7) such as mean dissolution time (MDT), area under the curve (AUC) and efficiency (E) were calculated. CSP-CTS/P407 presents the lowest value of MDT, 1.7 h, compared to 2.9 and 3.2 h of CSP-P407 and CSP-CTS, respectively. This fact indicates that among three formulations, one of them is able to release the drug most rapidly and it could provide better bioavailability in the tissue. The AUC results are statistically different, being lower for CSP-CTS, 2660.0 μg/h, and higher for CSP-P407, 14,785.3 μg/h. Regarding the results of efficiency, CSP-CTS/P407 shows the highest value, 78.6%. Probably, the interconnected porous morphology of CSP-CTS/P407 favors a better distribution of the drug within the hydrogel compared to the chains and agglomerated micelles of CSP-CTS and CSP-P407, respectively.

### 2.3. Ex Vivo Permeation of Caspofungin through Vaginal Mucosa

The vagina is a non-invasive route of administration in women that provides both a local and systemic effects. The aim of this study is to demonstrate that the therapeutic effect occurs locally in the vaginal mucosa. This is essential for fungal infections because fungi, mainly *Candida* spp., colonize the mucosal surface [67]. Therefore, a certain degree of permeation and retention of the drug in the tissue is necessary without reaching or exceeding systemic therapeutic levels, *C_min_*= 3.55 µg/mL and *C_max_*= 11.73 µg/mL [68,69,70]. Porcine and human vaginal mucosas are substantially similar. Hence, porcine vaginal mucosa was used for permeation studies [57]. There are not previous studies reporting permeation parameters of CSP through vaginal porcine mucosa.

**Figure 8:**
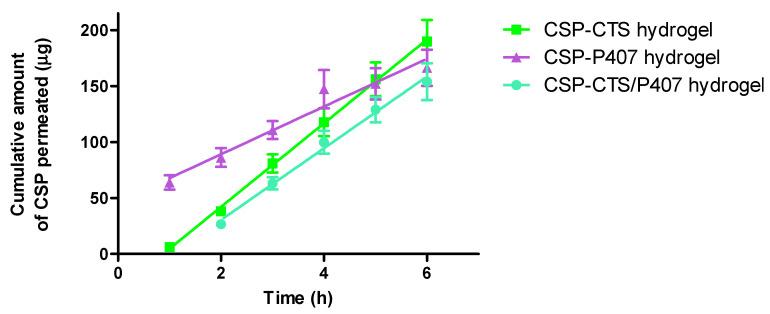
Cumulative amount of CSP permeated (μg) through vaginal mucosal upon application of CSP hydrogels. Each value represents the mean ± SD (*n* = 3).

Figure 8 illustrates the permeation profiles of CSP hydrogels through vaginal mucosa. The highest permeation profile was found for CSP-CTS hydrogel, followed by CSP-P407, and finally, CSP-CTS/P407. CTS has the capacity to interact with epithelial tight junctions increasing drug penetration [63,71]. Additionally, P407 is a potential enhancer for transmucosal drug delivery [72], due to their surfactant properties [73]. Interestingly, CSP-CTS/P407 hydrogel showed the lowest absorption, and will predictably produce the fewest side effects.

The calculated permeation kinetics parameters and CSP retained amount are reported in Table 4. Taking into account an application surface area of 62.8 cm^2^ for the vaginal cavity [74], and a plasmatic clearance (*Cl_p_*) of 0.55 L/h [68,69,70], the theoretical human plasmatic steady-state concentration (*Cl_ss_*) that CSP would achieve was also calculated, being 1.65 ± 0.01 μg/mL, 0.95 ± 0.22 μg/mL and 1.43 ± 0.12 μg/mL for CSP-CTS, CSP-P407 and CSP-CTS/P407, respectively. Therefore, no systemic side effects related to the use of CSP are expected. Finally, the amount of CSP remaining in the vaginal mucosa for CSP-CTS, CSP-P407 and CSP-CTS/P407, resulted to be 156.23 μg/cm^2^, 118.51 μg/cm^2^ and 409.40 μg/cm^2^, respectively.

To obtain the maximum therapeutic efficacy of the antifungal treatment, the formulation must remain long enough and at high concentration in the vaginal epithelium [75]. Based on the obtained data, the proposed formulation that is able to penetrate and retain significant amounts of CSP in the vaginal mucosa is CSP-CTS/P407 hydrogel. Then this is the best formulation to ensure a local action. 

### 2.4. Histological Evaluation

**Figure 9:**
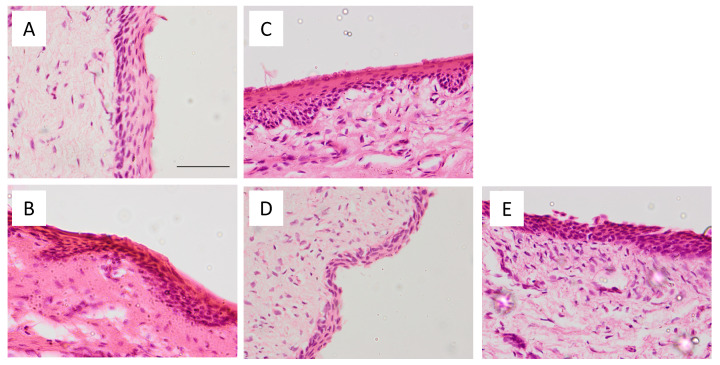
Histological sections of vagina stained with haematoxylin and eosin of: (**A**) normal vaginal mucosa; (**B**) damaged vaginal mucosa with ethanol; (**C**) vaginal mucosa treated with CSP-CTS hydrogel; (**D**) vaginal mucosa treated with CSP-P407 hydrogel; and (**E**) vaginal mucosa treated with CSP-CTS/P407 hydrogel. Scale bar = 50 μm, magnification = 400×.

The histomorphological analysis of the vaginal mucosa was performed under light microscope. Representative images of assayed treatments are shown in Figure 9. The non-keratinized stratified squamous vaginal epithelium (about 6–7 layers of cells) can be shown in control conditions (Figure 9A). A treatment with ethanol was performed as a positive control to show altered epithelium. The ethanol only induced a contraction of the epithelium. The treatment with CSP-CTS and CSP-CTS/P407 (Figure 9C,D, respectively) conserved the thickness of the epithelium whereas the treatment with CSP-P407 hydrogel (Figure 9E) induced the loss of some of the outermost layers during the processing of the samples. 

### 2.5. Antifungal Efficacy

The antimicrobial activity study of the CSP-loaded hydrogels has shown that they are effective on different species of *Candida*, producing wide areas of inhibition, and demonstrating the effectiveness of these products. Although for *C. auris* DSM 21092 they strongly inhibit after 24 h of incubation, a small resistant growth was found in the initial inhibition area at 48 h and, consequently, they would not be useful to eliminate this strain.

In the case of blank formulations (CTS, P407 and CTS/P407 hydrogels without CSP), it was observed that they prevent the development of studied yeasts at the point where samples were applied on the agar plate. These formulations contain CTS, which has been reported to posses antimicrobial activity against a wide range of microorganisms, such as bacteria, fungi, and yeast [35,76,77]. The formulations did not allow development at the point of application and did not diffuse into the agar surface as the CSP-loaded hydrogels did. An inhibitory action of CTS on fungal growth was evidenced, but it seems as previously described [78] that it largely depends on the physicochemical characteristics.

The results obtained are summarized in Table 5. CSP-CTS, CSP-P407 and CSP-CTS/P407 hydrogels would be useful for the treatment of VVC, as they have been effective in eliminating four of the *Candida* spp. tested.

## 3. Conclusions

In this work, CSP-loaded hydrogels of CTS, P407 and combination of both have been developed for the first time aimed for the treatment of VVC. All formulations were extensively characterized. The CSP-CTS/P407 hydrogel, formed by the combination of both polymers, proved to be the best formulation for topical administration of CSP against fungal infections. This hydrogel showed better spreadability and extrudability properties than the reference formulation, important factor to evaluate in topical application. It has excellent mucoadhesive properties, essential for formulations destined for vaginal drug administration. In addition, it was shown to be the formulation that retained the highest amount of CSP in the vaginal mucosa, providing the desired local therapeutic effect. It should therefore not cause any systemic side effects. The hydrogel was well tolerated by the vaginal mucosa because no significant mucosal barrier disruption or irritability was observed. These results demonstrate that CSP-CTS/P407 hydrogel is a stable, suitable and safe formulation for the treatment of VVC. 

## 4. Materials and Methods

### 4.1. Materials

#### 4.1.1. Chemical and Reagents

CSP acetate salt was supplied from SunPharma (Barcelona, Spain). Trifluoroacetic acid, methanol, mucin from porcine stomach type III and medium molecular weight CTS (190–310 KDa molecular weight, ≥75% deacetylated) were purchased from Sigma Aldrich (Madrid, Spain). Pluronic^®^ F-127 (P407) was provided by BASF (Barcelona, Spain). Acetic acid was obtained from Panreac (Barcelona, Spain). Physiological saline 0.9% was supplied by Grifols Laboratories S.A (Barcelona, Spain). Double distilled water was used after filtration in a Milli-Q^®^ Gradient A10 system apparatus (Millipore Iberica S.A.U.; Madrid, Spain). Commercial formulation of clotrimazole (Gine-Canestén^®^, 20 mg/g) for vaginal application was obtained from a local pharmacy. Ketamine HCl was purchased from Pfizer (Madrid, Spain). Xylazine was obtained from Laboratories Calier (Barcelona, Spain). Midazolam, propofol and sodium thiopental were supplied by B. Braun Medicals (Barcelona, Spain). Isoflurane was purchased from Baxter (Valencia, Spain). Hank’s balanced salt solution (HBSS) (Composition in g/L: CaCl_2_ = 0.14; KCl = 0.14; KH_2_PO_4_ = 0.06; MgSO_4_ = 0.1; MgCl_2_ = 0.1; NaCl = 8.0; NaHCO_3_ = 0.35; Na_2_HPO_4_ = 0.09; Glucose = 1) was obtained from Biological Industries (Kibbutz Beit Haemek, Israel). Polytetrafluoroethylene (PTFE) membranes were acquired from Pall^®^ Corporation (Madrid, Spain). All other chemicals and reagents used in this study were all of analytical grade.

#### 4.1.2. Biological Tissue for Ex Vivo Assays

Vaginal mucosa tissues were obtained from pigs in accordance with protocols prescribed by the Animal Experimentation Ethics Committee of the University of Barcelona, Spain (CEEA-UB) code 10617. Female pigs weighing 20–25 kg, were anesthetized with intramuscular administration of ketamine HCl (3 mg/kg), xylazine (2.5 mg/kg) and midazolam (0.17 mg/kg). Propofol (3 mg/kg) was administered via atrial vein after sedation and immediately afterwards they were intubated and maintained under anesthesia inhaled with isoflurane. Finally, three animals were sacrificed under veterinary supervision at the Animal Facility of the Bellvitge Campus of the University of Barcelona (Spain) using an overdose of sodium thiopental. Vaginal mucosa tissues were maintained in HBSS and refrigerated until experiments [79]. Before use, the vaginal mucosa tissues were superficially cleaned with gauze soaked in 0.05% dodecyl sulphate solution, followed by distilled water.

### 4.2. Preparation of CSP Hydrogels

Firstly, blank CTS hydrogel was prepared by dispersion of appropriate amount of CTS (2%, *w*/*v*) in 1% aqueous acetic acid, previously prepared, with continuous stirring until homogenization. Subsequently, CSP loaded CTS hydrogel (CSP-CTS hydrogel) was obtained by solubilization of CSP in blank CTS hydrogel by manual stirring, yielding at 2% (*w*/*v*) hydrogel of CTS and CSP.

CSP loaded P407 hydrogel (CSP-P407 hydrogel) was elaborated as follows; a concentration of 20% (*w*/*v*) P407 and 2% (*w*/*v*) CSP were mixed and stirred overnight at 4 °C.

**Figure 10:**
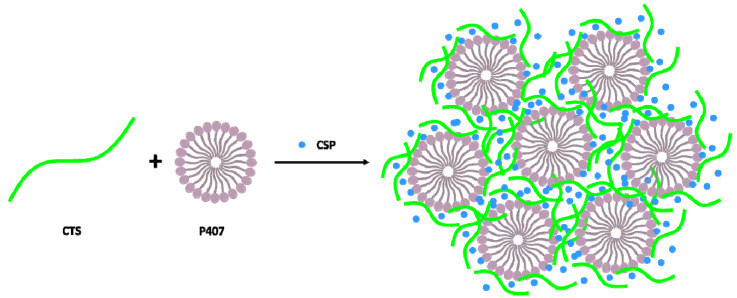
Schematic representation of the formation of the CSP-CTS/P407 hydrogel.

On the other hand, to prepare CSP loaded CTS/P407 hydrogel (CSP-CTS/P407 hydrogel), an appropriate amount of P407 and CSP was pre-mixed and kept at 4 °C. This mixture was dispersed in a pure CTS hydrogel to reach a final concentration of 2% (*w*/*v*) for CTS and CSP, and 20% (*w*/*v*) for P407. The formation of this hydrogel is represented schematically in Figure 10.

After preparation of hydrogels no insoluble particles were observed. Therefore, all samples were stored at 4 °C.

### 4.3. Physical Characterization of CSP Hydrogels

#### 4.3.1. pH Measurements

The determination of the pH values of samples was performed using a digital pH/mV-meter-pH 200 (Crison Instruments S.A., Barcelona, Spain). Measurements of samples were conducted at 25 °C with the hydrogel freshly prepared and stored in a refrigerator (4.00 ± 1.00 °C) two months after preparation. Readings were recorded as the mean ± standard deviation (SD) of three replicates.

To mimic application conditions, the formulations were mixed with SVF, which was prepared by mixing NaCl 3.510 g, KOH 1.400 g, Ca(OH)_2_ 0.220 g, bovine serum albumin 0.018 g, lactic acid 2.000 g, acetic acid 1.000 g, glycerol 0.160 g, urea 0.400 g and glucose 5.000 g to 900 mL of distilled water in a beaker, and stirred until completely dissolved. The pH of the mixture was then adjusted to 4.5 using HCl, and the final volume was adjusted to 1 L [50,80].

#### 4.3.2. Morphological Studies

To analyze the structure of developed formulations a JEOL JSM-7001F scanning electron microscope (JEOL Ltd., Peabody, MA, USA) was used. The CSP hydrogels were dried for 5 days using a temperature-controlled heater. Once they have dried, a small quantity of each sample was fixed on a double-coated carbon conductive tape and coated with a thin layer of carbon as a conductor agent in an Emitech K950 coater (Quorum Technologies Ltd., Kent, UK). Finally, the samples were observed in high vacuum mode.

#### 4.3.3. Surface Electrical Properties

The surface electrical properties were assessed based on the method previously described by Shaarani et al. [53]. The ZP measurements of unloaded and loaded hydrogels were performed at 25 ± 0.5 °C using a Zetasizer^®^ 2000 (Malvern Instruments Ltd., Worcestershire, UK). Before measuring, the samples were diluted 100 times in double distilled water. Values are reported as the mean ± SD of nine replicates.

#### 4.3.4. Rheological Behavior

The rheological characterization of the samples was performed 24 h after hydrogels preparation using a Haake RheoStress 1 rheometer connected to a temperature control Thermo Haake Phoenix II + Haake C25P (Thermo Fisher Scientific, Karlsruhe, Germany). It was equipped with a parallel plate-plate geometry set-up including a fixed bottom plate and a Haake PP60Ti movable upper plate (60 mm diameter) and operated using Haake RheoWin^®^ Job Manager and Data Manager software v. 4.87.

Rotational tests were carried out to determine the viscosity and flow behavior of the formulations. Each sample was equilibrated by placing it between the plate-plate sensor system (0.5 mm gap) for 5 min to attain the running temperature (4, 25 and 37 °C). Then, they were submitted to a three steps shear profile program: a ramp-up period (0–50 s^−1^) for 3 min, constant shear rate period at 50 s^−1^ for 1 min and a ramp-down period (50–0 s^−1^) for 3 min. The data from the flow curves (shear stress (τ) versus shear rate (Υ) were fitted to different mathematical models (Newton, Bingham, Ostwald-de-Waele, Herschel-Bulkley, Casson and Cross) [81,82]. The model that statistically best described the experimental data was selected based on the best correlation coefficient value (r).

The apparent thixotropy was determined by the presence or the absence of a hysteresis loop. Viscosity mean values (Pa·s) were determined at 50 s^−1^ from the constant share rate period of each viscosity curve (viscosity (η) versus shear rate (Υ).

In addition, the thermosensitive gelation process of the CSP-P407 hydrogel was investigated upon temperature sweeping from 10 to 40 °C under oscillation mode with a fixed frequency of 1 Hz and a constant stress of 0.5 Pa. Each sample was equilibrated by placing it between the plate-plate system for 5 min to attain the start temperature of 4 °C. Then the temperature was increased from 10 to 40 °C at a controlled ramp speed for 2000 s and the modules G′ and G″, as well as complex viscosity (η*) were measured.

The temperature at which a sharp rise in viscosity was detected (coinciding with the crossover point where G’ = G’’) was recorded as gelation temperature or SOL-GEL temperature transition (TSOL-GEL) [83,84].

#### 4.3.5. Spreadability Test

The extensibility test was evaluated for the CSP-CTS, CSP-P407, CSP-CTS/P407 hydrogels using a commercial formulation of clotrimazole, Gine-Canestén^®^ (20 mg/g) as reference. To evaluate this property, 0.5 g of each sample was placed within a circle pre-marked on a glass plate over which a second glass plate was overlapped, as focused as possible, without sliding of the plates. Force was generated onto the upper plate by adding known weights (5, 10, 20, 30, 40, 50, 100, 150 and 200 g). So, the samples were compressed to uniform thickness. The weights were removed after 60 s and the area was recorded. The results were expressed in terms of the spreading area as a function of the applied mass according to the following equation:

S = d^2^ × π/4 (1)

where S is the spreading area (cm^2^) resulting from the applied mass (g), and d is the mean diameter (cm) reached by the sample. Each sample was measured in triplicate for each weight at room temperature. 

Finally, the spreadability results were fitted to mathematical equations (Boltzmann, hyperbola one site and hyperbola two sites). The goodness of the fitting was confirmed by r value.

#### 4.3.6. Extrudability Study

**Figure 11:**
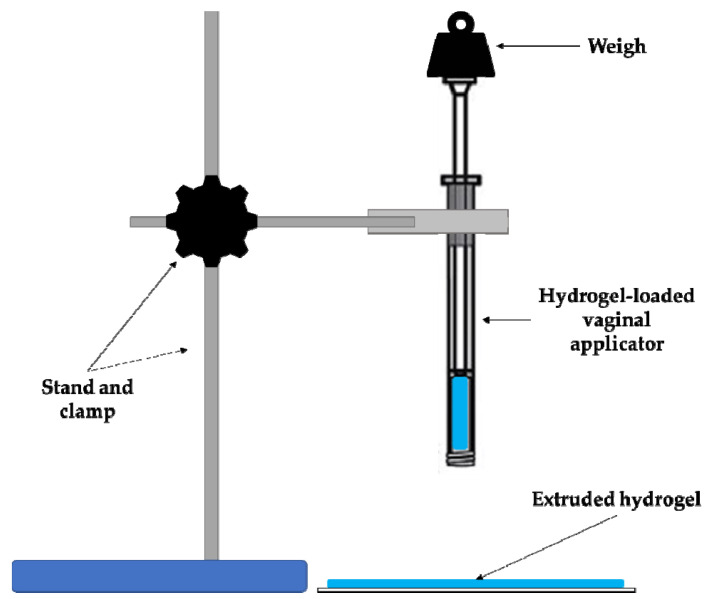
Schematic illustration of the device used for evaluation of spreadability study.

This test measures the required force to remove the hydrogel from the container. The syringeability of the developed hydrogels was measured in terms of the weight necessary to be applied to remove a known amount of hydrogel from a vaginal applicator (Figure 11). 

For that purpose 5 g of each formulation was carefully loaded into vaginal applicators avoiding the formation of air bubbles. The device was vertically placed on a support and known weights were added to its plunge. The plunger was pressed down and the extruded weight was recorded. The system was maintained at 4 ± 0.50 °C, to simulate application conditions. 

The syringeability of developed CSP hydrogels was calculated according to the following equation:

E = W/A(2)

where W is the weigh applied (g) to extrude the sample from the applicator and A the area (cm^2^) of the extruded hydrogel from the vaginal applicator. The data obtained were expressed as the mean ± SD of three replicates.

#### 4.3.7. Evaluation of Mucoadhesion Properties

The mucoadhesive property of CSP formulations was evaluated by measuring the influence of the samples on the zeta potential of mucin [85]. Each sample was dispersed with an equivalent amount of porcine mucin solution 1% (*w*/*v*) under continuous stirring. The mixtures of hydrogel-mucin were allowed to equilibrate overnight at room temperature. The surface charge of samples was detected using a Zetasizer^®^ 2000 (Malvern Instruments Ltd., Worcestershire, UK). Readings were recorded as the mean ± SD of nine replicates.

### 4.4. In Vitro Release and Kinetic Evaluation

The in vitro release study of CSP from the hydrogels was performed using amber glass vertical diffusion Franz cells (FDC 400, Crown Glass, Somerville, NY, USA) of 12 mL volume receiver chamber and 2.54 cm^2^ effective diffusional area. Polytetrafluoroethylene membranes (wwPTFE 0.45 µm 47 mm discs) were mounted between the donor and receptor compartment. The receptor fluid consisted of physiological saline 0.9%, which was kept under continuous stirring (600 rpm). The system was maintained at 37 ± 0.50 °C, to simulate vaginal conditions in vivo. 

Drug release studies were carried out by placing a known amount of CSP hydrogels on the donor compartment. Air bubbles under the membranes were removed and the system was allowed to equilibrate for at least 30 min before samples were applied. To prevent evaporation of the donor compartment and sampling ports, these were sealed with parafilm. At different scheduled times during 8 h, samples of receptor compartment were collected and the same volume was immediately replaced with tempered physiological saline. The collected samples were analyzed for CSP content by Ultra Performance Liquid Chromatography (UPLC) methodology previously validated according to ICH Q2 (R1) guidelines. Values are reported as the mean ± SD of three replicates. The data obtained were fitted to various mathematical models (One phase exponential association, Boltzmann sigmoidal, one site binding) to determine the release kinetics. The model was chosen according to the best r^2^ value.

Furthermore, some amodelistic parameters such as mean dissolution time (MDT), the area under the curve (AUC), and efficiency (E) were also estimated. MDT is the mean dissolution time of the CSP in the formulation along the process. AUC represents the amount of CSP that is released from the formulation and E the efficiency.

### 4.5. Ex Vivo Permeation of CSP through Vaginal Mucosa

The ex vivo drug permeation study of CSP was carried out using amber glass vertical diffusion Franz cells (FDC 400, Crown Glass, Somerville, NY, USA). Porcine vaginal mucosas were used as permeation membranes with the epithelium region facing the donor compartment and the connective region facing the receiver compartment. The receptor compartments were filled with physiological saline 0.9%, which were kept under continuous stirring at 600 rpm and maintained at 37 ± 0.5 °C. Sink conditions were ensured throughout the study. At different scheduled times samples of receptor compartment were collected via syringe and the same volume was immediately replaced with physiological saline for 6 h. The collected samples were analyzed for drug content by UPLC. Three determinations were made for each sample from the same donor to avoid variability due to membranes. Values are reported as the mean ± SD.

#### 4.5.1. Calculation of the Permeation Parameters

The cumulative amounts of CSP (µg) that had penetrated per unit of vaginal mucosa surface area (cm^2^) were collected and plotted versus time (h). Permeation parameters as flux (J, μg/h) was calculated by plotting the cumulative amount of permeated drug against time by using the Prism^®^ software v. 5.00 (GraphPad Software Inc., San Diego, CA, USA). The slope of the linear regression was also determined. 

The permeability coefficients (K_p_, cm/h) were obtained according to the following equation:

K_p_ = J/C_0_ (3)

where C_0_ is the initial drug concentration in the donor compartment, 20,000 μg/mL.

The predicted drug steady-state plasma concentration (C_ss_, μg/mL) that would penetrate after topical application was calculated using the equation:

C_ss_ = J × A/Cl_p_ (4)

where C_ss_ is the plasma steady-state concentration, J the flux determined in this study, A the hypothetical area of application (62.8 cm^2^) and Cl_p_ the plasmatic clearance (0.55 L/h) [68,69,70].

Finally, the data obtained from permeation studies as a function of time were fitted to different mathematical models to determine the kinetics. 

#### 4.5.2. Amount of CSP Retained in the Vaginal Mucosa

In order to detect differences between formulations, the quantity of CSP remaining on the vaginal mucosal, Q_r_ (µg/cm^2^), was also determined. For this, after permeation assays, the residual CSP hydrogel on the vaginal mucosa membranes was removed using a swab. The tissues were taken out of the device and cleaned with gauze soaked in sodium lauryl sulphate 0.05% and washed with distilled water. The permeation areas of the vaginal mucosa membranes were cut, weighed, perforated with a thin needle and immersed in 2 mL of physiological saline 0.9% for 15 min using an ultrasonic bath. Samples were analyzed by UPLC. 

### 4.6. Analysis of CSP in Solution

The UPLC system consisted in an Acquity I CLASS UPLC System (Waters, Milford, CT, USA) coupled to an Acquity TUV and an Acquity Fluorescence Detector. Fluorescence detection was carried out at 224 and 304 nm for excitation and emission wavelengths, respectively. 

The chromatography separation was performed with a Lichrospher RP-8 column (125 × 4 mm, 5 µm, Phenomenex). The method had the column at room temperature. The flow rate and the injection volume were 0.8 mL/min during 8 min and 10 µL, respectively. The mobile phase A consisted of 0.1 % of trifluoroacetic acid (TFA) in Milli-Q^®^ water and mobile phase B comprised methanol. The gradient elution program was: 50–50% B for 0–5 min; 100% B for 5–6 min; 50–50% B for 6–8 min.

### 4.7. Histological Evaluation

The hydrogel formulations were applied for 6 h to study the vaginal mucosal. Then, the tissue was removed from the Franz cell rinsed with PBS pH 7.4, and set overnight in 4% buffered formaldehyde and embedded in paraffin wax. Transversal sections (6 µm) were stained with hematoxylin and eosin and observed for the evaluation of the histomorphology under a light microscope Olympus BX41 equipped with Olympus XC50 camera (Olympus Co., Tokyo, Japan). Tissues without gel (blank vaginal mucosa) and with ethanol (damaged vaginal mucosa) were used as the control and positive control conditions, respectively.

### 4.8. Antifungal Efficacy

Antifungal activity of developed formulations was tested by using a modification of the Kirby-Bauer disk diffusion test [86]. Basically, the methodology described in the Test Protocol [87] was followed without using disk, but using the capacity to diffuse around the place of the agar where product has been deposited. Five strains of *Candida* were used to study antimicrobial activity: *Candida albicans* ATCC 10231 (American Type Culture Collection, Manassas, VA, USA), *Candida auris* DSM 21092 (German collection of Microorganisms and cell cultures GmbH), *Candida tropicalis* ATCC 7349, *Candida glabrata* ATCC 66032 and *Candida parapsilosis* ATCC 22019. The yeast strains were first grown aerobically in Mueller-Hinton (MH) medium supplemented with glucose 2% at 30 °C for 48 h. The fungi inoculums were prepared by suspending colonies in Ringer’s solution to achieve the desired density equivalent to the 0.5 McFarland. MH-glucose 2% plates were inoculated three times over the entire agar surface with the help of a yeast-soaked swab by streaking action, rotating the plate approximately 60° each time to ensure an even distribution of the inoculum that result in a confluent lawn of growth.

The following formulations were studied: CSP-CTS, CSP-P407 and CSP-CTS/P407 hydrogels; CTS, P407 and CTS/P407 hydrogels and controls of nystatin 100 UI/mL and amphotericin B 250 µg/mL. About 5 µL of these products were placed onto de yeast inoculated MH-glucose 2% agar. Plates were incubated at 30 °C during 48 h. The zone of inhibition for growth of yeasts was observed. Formulations CTS, P407 and CTS/P407 hydrogels were also tested in a MH–glucose 2% medium where 500 µg/mL chloramphenicol was added to avoid possible bacterial contamination on excipients.

### 4.9. Statistical Analysis

Experimental data were statistically evaluated by one-way analysis of variance (ANOVA) using the GraphPad Prism^®^ software v. 5.00 (Graphpad Software Inc., San Diego, CA USA). Differences were considered significant at *p* < 0.05 level. To determine the differences between independent groups, Tukey’s test was applied, adopting a confidence level of 95%.

## Figures and Tables

**Table 1:** Zeta potential data of unloaded and loaded formulations. Zeta potential data represent the mean ± SD of nine replicates.

	Zeta Potential Values (mV)	
	CSP-Loaded	CSP-Unloaded
CTS hydrogel	80.36 ± 4.72	74.00 ± 4.19
P407 hydrogel	11.14 ± 1.62	9.94 ± 0.74
CTS/P407 hydrogel	49.76 ± 3.22	40.51 ± 3.87

**Table 2:** Results of the rheological rotational testing of the formulations CSP-CTS, CSP-P407 and CSP-CTS/P407 hydrogels.

Samples	Temperature (°C)	Rheological Behavior and Model		Viscosity at 50 s^−1^ (mPa·s)	Apparent Thixotropy
		Stretch Ramp-Up	Stretch Ramp-Down		
CSP-CTS hydrogel	4	Pseudoplastic (Cross, r = 0.9998)	Pseudoplastic (Cross, r = 1)	2237 ± 13.40	Presence
	25	Pseudoplastic (Cross, r = 0.9999)	Pseudoplastic (Cross, r = 1)	1645 ± 11.87	Presence
	37	Pseudoplastic (Cross, r = 0.9999)	Pseudoplastic (Cross, r = 1)	1291 ± 7.08	Presence
CSP-P407 hydrogel	4	Pseudoplastic (Cross, r = 0.9997)	Newtonian (Newton, r = 0.9999)	16.45 ± 0.60	Absence
	25	Irregular flow	Irregular flow	73.94 ± 6.09	Presence
	37	Pseudoplastic (Cross, r = 0.9989	Pseudoplastic (Cross, r = 0.9974)	70.73 ± 2.02	Presence
CSP-CTS/P407 hydrogel	4	Pseudoplastic (Cross, r = 0.9997)	Pseudoplastic (Cross, r = 1)	1868 ± 14.11	Presence
	25	Pseudoplastic (Cross, r = 0.9998)	Pseudoplastic (Cross, r = 1)	1425 ± 12.88	Presence
	37	Pseudoplastic (Cross, r = 0.9997)	Pseudoplastic (Cross, r = 1)	1009 ± 5.51	Presence

**Table 3:** Extrudability (g/cm^2^) behavior at 4 °C of reference and CSP hydrogel. Each value represents the mean ± SD (*n* = 3).

	Extrudability (g/cm^2^)
**Reference**	8.05 ± 0.29 *
CSP-CTS hydrogel	4.52 ± 0.43 *
CSP-P407 hydrogel	0.07 ± 0.01 *
CTS/P407 hydrogel	1.83 ± 0.09 *

* Represent significant differences (*p* < 0.001).

**Table 4:** Estimated permeation and retention parameters of CSP hydrogels. Values are reported as the mean ± SD (*n* = 3).

Biopharmaceutical Parameter	Formulations			Statistic
	CSP-CTS Hydrogel ^1^	CSP-P407 Hydrogel ^2^	CTS/P407 Hydrogel ^3^	*p* < 0.05
J (μg/h)	36.76 ± 0.31	21.08 ± 4.98	31.84 ± 2.76	1 vs. 2 2 vs. 3
K_p_ × 10^4^ (cm/h)	7.24 ± 0.06	4.15 ± 0.98	6.27 ± 0.54	1 vs. 2 2 vs. 3
C_ss_ (μg/mL)	1.65 ± 0.01	0.95 ± 0.22	1.43 ± 0.12	1 vs. 2 2 vs. 3
Q_r_ (µg/cm^2^)	156.23 ± 14.91	118.51 ± 12.78	409.40 ± 48.34	1 vs. 3 2 vs. 3

**Table 5:** Antimicrobial activity of CSP-CTS, CSP-P407 and CSP-CTS/P407 hydrogels; and CTS, P407 and CTS/P407 hydrogels.

	Yeast Tested				
	*C. albicans* ATCC 10231	*C. auris* DSM 21092	*C. tropicalis* ATCC 7349	*C. glabrata* ATCC 66032	*C. parapsilosis* ATCC 22019
Production of large areas of inhibition					
CSP-CTS hydrogel	+ ^(a)^	R ^(b)^	+	+	+
CSP-P407 hydrogel	+	R	+	+	+
CSP-CTS/P407 hydrogel	+	R	+	+	+
Inhibition of fungi in situ where the sample has been deposited					
CTS hydrogel	+r ^(c)^	+r	+	+	+
P407 hydrogel	+	+	+	+	−
CTS/P407 hydrogel	+r	+r	+	+	+

^(a)^ +; formulation totally effective against the strain tested; − formulation does not produce any type of action on yeast growth. ^(b)^ R; There is a very marked inhibition effect on yeast growth at 24 h, but when incubating for longer up to 48 h, resistant growth appears within the first inhibition zone. ^(c)^ +, − o +r; Regarding the antifungal activity of the excipients at the point where the sample has been placed, whether they have inhibited growth, +, or not, −, or inhibit with resistant growth, +r.

## Data Availability

The data presented in this study are available on request from the corresponding author. The data are not publicly available due to they are part of a Doctoral Thesis and it will be available once the Thesis will be published.
